# Beyond Improvement of Motor Symptoms: Central Effects of Botulinum Toxin on Anxiety and Depression in Focal Dystonia, Hemifacial Spasm, and Blepharospasm

**DOI:** 10.3390/toxins18020062

**Published:** 2026-01-25

**Authors:** Tihana Gilman Kuric, Zvonimir Popovic, Sara Matosa, Eleonora Strujic, Ivana Gacic, Tea Mirosevic Zubonja, Stjepan Juric, Melita Pecek Prpic, Vera Jelusic, Dubravka Biuk, Svetlana Tomic

**Affiliations:** 1 Department of Neurology, Osijek University Hospital Center, 31000 Osijek, Croatia; 2 Faculty of Medicine in Osijek, Josip Juraj Strossmayer University of Osijek, 31000 Osijek, Croatia; 3 General Hospital “Dr. Josip Benčević” Slavonski Brod, 35000 Slavonski Brod, Croatia; 4 Osijek-Baranja County Health Centre, 31000 Osijek, Croatia; 5 Department of Ophthalmology and Optometry, Osijek University Hospital Center, 31000 Osijek, Croatia

**Keywords:** botulinum neurotoxin type A (BoNT-A), cervical dystonia (CD), blepharospasm, hemifacial spasm (HFS), mood modulation

## Abstract

Cervical dystonia (CD), blepharospasm (BSP), and idiopathic hemifacial spasm (HFS) are focal hyperkinetic movement disorders with distinct underlying mechanisms. While CD and BSP involve central network dysfunctions within the basal ganglia-thalamo-cortical and cerebellar circuits, HFS primarily results from peripheral facial nerve hyperexcitability. Still, people living with all three conditions often struggle with mood issues like depression and anxiety, which can originate from both the burden of illness and changes in brain biology. We studied 61 patients (CD, *n* = 30; BSP, *n* = 9; HFS, *n* = 22) and assessed depression and anxiety before and three weeks after botulinum neurotoxin type A (BoNT-A) therapy, considering injection site and dose. BoNT-A significantly reduced depressive and anxiety symptoms across all groups, regardless of disease type, dose, or glabellar injection. These psychiatric improvements were not associated with the degree of motor symptom reduction, suggesting a partially independent mechanism of mood modulation. Our findings indicate that BoNT-A’s mood benefits may extend beyond local motor effects, possibly involving broader sensorimotor-limbic interactions. These results highlight the therapeutic potential of BoNT-A for addressing non-motor symptoms in both dystonic and non-dystonic hyperkinetic disorders. Future studies employing imaging and neurophysiological methods are necessary to explain the neural pathways underlying these effects.

## 1. Introduction

Focal dystonias such as cervical dystonia (CD) and blepharospasm (BSP) are hyperkinetic movement disorders characterized by sustained or intermittent muscle contractions that lead to abnormal postures or movements [1]. Although traditionally regarded as purely motor conditions, increasing evidence supports a broader concept in which non-motor symptoms, particularly anxiety and depression, represent intrinsic features of these disorders rather than secondary psychological reactions [2,3]. Various studies have documented high rates of affective symptoms in CD and BSP that do not correlate with motor severity, suggesting shared neurobiological mechanisms [4,5]. Neuroimaging findings further reveal abnormal connectivity between the amygdala, anterior cingulate cortex, insula, and sensorimotor networks, implicating limbic–motor dysfunction as a key contributor to both motor and emotional symptoms [6,7].

Botulinum neurotoxin type A (BoNT-A) remains the first-line treatment for focal dystonias, offering reliable improvement in motor symptoms [8], with accumulating evidence indicating it is extended therapeutic effect beyond the neuromuscular junction [9,10]. Preclinical animal studies further support this concept, demonstrating that BoNT-A can modulate central neurotransmission, alter afferent sensory processing, and influence limbic-striatal circuits through mechanisms such as retrograde transport and synaptic modulation [11,12].

Several studies, including our previous work, have demonstrated significant reductions in depressive and anxiety symptoms following BoNT-A therapy in patients with cervical dystonia, even when these improvements were not directly related to motor response [9,13]. These findings suggest central or network-level mechanisms, potentially mediated by modulation of somatosensory feedback or by altered connectivity within cortico-limbic circuits [14].

Complementary evidence arises from psychiatric research on BoNT-A injections in the glabellar region, which targets the corrugator and procerus muscles involved in frowning. According to the facial feedback hypothesis, decreased proprioceptive input from these muscles may influence affective processing [14,15]. Randomized controlled trials and neuroimaging studies demonstrate that glabellar BoNT-A injections alleviate depressive symptoms and modify amygdala reactivity, supporting a direct influence on emotional regulation pathways [16,17].

The amygdala, central to emotion-motor integration, has been implicated in CD, BSP, and even hemifacial spasm (HFS), a peripheral (and idiopathic) hyperkinetic disorder lacking dystonic pathophysiology. In HFS, emotional stress commonly exacerbates symptoms, and resting-state neuroimaging demonstrates altered amygdala activity and connectivity, suggesting that central limbic circuits may play a role in symptom modulation [18].

From this perspective, we aimed to compare changes in depression and anxiety in patients with CD, BSP, and HFS following BoNT-A therapy, to determine whether mood improvement depends on injection site and botulinum toxin dosage. Demonstrating comparable affective benefits across all groups (including those without glabellar injections) would support the hypothesis that BoNT-A exerts central neuromodulatory effects on limbic networks, independent of local muscle activity or disease mechanism.

## 2. Results

### 2.1. Changes in Motor and Non-Motor Symptoms After BoNT-A

Botulinum toxin therapy was associated with a significant reduction in motor symptoms, pain, and the severity of depressive and anxiety symptoms (Table 1).

### 2.2. I Between-Group Comparison of Mood Changes After BoNT-A

Improvements in both BDI-II and BAI scores were observed across all diagnostic groups, with no significant differences in the magnitude of change between patients with CD, HFS, or BSP (Table 2). Although the numerical size of these changes varied between conditions, the differences were not statistically significant, likely reflecting within-group variability and unequal sample sizes. Overall, the therapeutic effect on mood and anxiety was consistent across conditions.

Changes in BDI-II and BAI scores did not differ between patients who received glabellar injections and those who did not (Table 3), indicating that mood improvement was not dependent on injection site.

**Figure 1:**
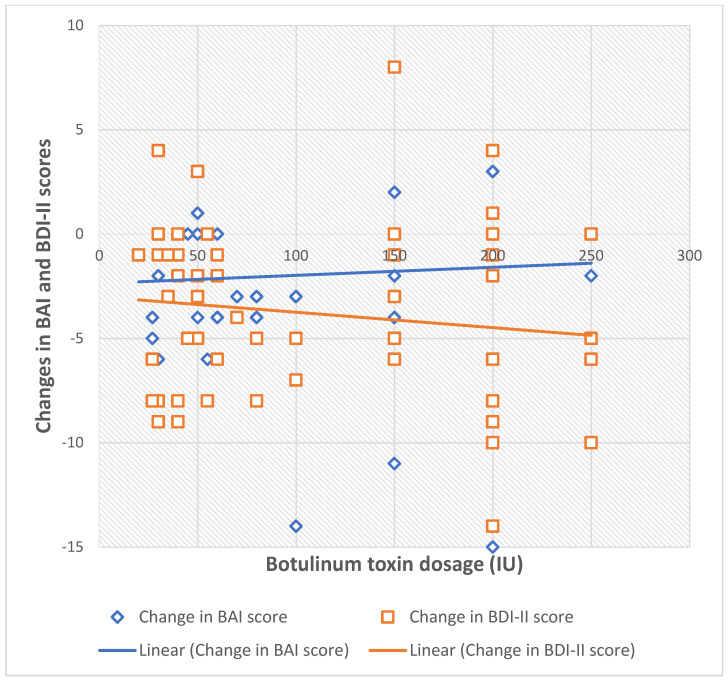
Scatterplot of BoNT’A dosage versus change in BDI’II and BAI scores, showing individual patient data and illustrating the absence of a dose–response relationship.

Doses of applied botulinum toxin show no correlation to changes in BDI-II (rho = 0.069, *p* = 0.60, Spearman’s Correlation) and BAI scores (rho = 0.023, *p* = 0.859, Spearman’s Correlation), respectively. To visually support the finding that BoNT-A dosage does not correlate with mood improvement, individual patient data are presented in the scatterplot below (Figure 1).

## 3. Discussion

The present study shows that BoNT-A treatment is associated with significant and comparable improvements in symptoms of depression and anxiety in patients with cervical dystonia (CD), blepharospasm (BSP), and hemifacial spasm (HFS). This is somewhat unexpected given the clear differences in the underlying mechanisms of these conditions, which range from central network dysfunction in dystonias to facial nerve hyperexcitability in idiopathic HFS. HFS is most often linked to vascular compression of the facial nerve or to increased excitability at the level of the brainstem. However, recent neuroimaging studies suggest that HFS may not be purely peripheral. Altered connectivity between motor and limbic regions has been reported [19], and there is growing interest in the possible contribution of neuroinflammatory processes [20].

Despite these differences in pathophysiology, patients across all three diagnostic groups showed a similar degree of improvement in affective symptoms after BoNT-A treatment. These findings suggest that the effects of BoNT-A on mood cannot be explained by muscle relaxation alone. Instead, they point toward a broader mechanism that may involve central neuromodulatory effects, possibly through changes in sensorimotor feedback or limbic–motor network interactions, in addition to its well-established peripheral action at the neuromuscular junction.

In addition to these network-level considerations, it is important to distinguish the biochemical mechanism of BoNT-A from the underlying disease pathology. BoNT-A acts peripherally by cleaving the SNAP-25 protein at the presynaptic terminal, thereby blocking acetylcholine release at the neuromuscular junction. This mechanism is fundamentally different from the central pathophysiological processes that characterize dystonias and hemifacial spasm, which involve basal ganglia and brainstem circuit dysfunction as well as abnormal sensorimotor integration. Beyond its peripheral chemodenervation, BoNT-A also modulates afferent input from muscle spindles, which may contribute to improvements in pain, proprioception, and affective symptoms.

### 3.1. Evidence from Previous Studies and Facial Feedback Research

Our findings are consistent with previous studies, including our own earlier work [5], which demonstrated significant reductions in depressive and anxiety symptoms following BoNT-A therapy in CD patients, even when these improvements were not directly related to motor response [20]. Such evidence points toward central or network-level effects, possibly mediated by modulation of somatosensory feedback or altered connectivity within cortico-limbic circuits [21]. Complementary psychiatric research on BoNT-A injections in the glabellar region supports the facial feedback hypothesis, showing that decreased proprioceptive input from frowning muscles can influence affective processing [14,22,23]. Randomized controlled trials and neuroimaging studies further demonstrate that glabellar BoNT-A injections alleviate depressive symptoms and modify amygdala reactivity [23,24]. The amygdala, central to emotion–motor integration, has also been implicated in CD, BSP, and HFS, where emotional stress exacerbates symptoms and neuroimaging reveals altered amygdala activity and connectivity [18]. These converging findings strengthen the interpretation that BoNT-A exerts central neuromodulatory effects on limbic networks, beyond local muscle relaxation.

Furthermore, analysis based on treatment region revealed no significant differences in changes in BDI-II and BAI scores between patients whose protocol included glabellar injections and those treated in other clinically relevant regions. In the latter group, BoNT-A was applied to periorbicular, perioral, and cheek muscles in HFS and BSP, and to cervical and cranial muscles according to the Col-Cap concept in CD (Table 3). This suggests that the improvement in psychiatric symptoms following BoNT-A therapy is not limited to patients treated in the glabellar area, but rather represents a broader effect across different forms of focal hyperkinetic disorders.

The pattern of improvement observed in our study aligns with the well-established clinical profile of BoNT-A, which consistently demonstrates reductions in motor severity, pain, and functional impairment across focal dystonias. The decrease in anxiety and depressive symptoms observed here is also consistent with reports linking psychiatric improvement to both motor relief and the broader neuromodulatory effects of BoNT-A. Our findings, therefore, fit within the expected therapeutic spectrum and reinforce the robustness of BoNT-A effects across different cranial and cervical dystonias.

### 3.2. Pathophysiological Background and Emotional Dysregulation in Focal Hyperkinetic Disorders

Neuroimaging and neurophysiological studies consistently demonstrate that dystonia is characterized by both reduced and aberrant connectivity across key motor and limbic circuits. Network-level models describe depressed functional coupling within cerebello-basal ganglia pathways and between the basal ganglia and sensorimotor cortex [25,26], alongside maladaptive or misdirected connectivity involving the basal ganglia, cerebellum, sensorimotor cortex, and limbic regions such as the amygdala, insula, and anterior cingulate cortex [26,27]. These findings indicate that dystonia does not reflect a uniform loss of connectivity, but rather a complex pattern of hypoconnectivity in some circuits and aberrant, compensatory hyperconnectivity in others [25,27].

This altered limbic-motor interplay is thought to explain why many patients with dystonia experience not only motor symptoms but also emotional dysregulation [28,29].

Mood disorders, particularly depression and anxiety, occur in up to 70% of CD and BSP patients, often preceding motor onset and persisting even when motor symptoms are well controlled [30]. This temporal and clinical dissociation implies that affective symptoms are intrinsic to the disease process rather than secondary to disability or disfigurement. Some imaging and neurophysiological studies (particularly in functional movement disorders) have shown increased activity in limbic regions (e.g., amygdala) and altered connectivity between limbic and motor/prefrontal areas, suggesting that abnormal limbic-motor interactions may contribute to emotional dysregulation and abnormal motor control [31,32,33].

HFS, traditionally considered a peripheral hyperkinetic disorder most often associated with vascular compression of the facial nerve or idiopathic brainstem hyperexcitability [34], may also involve central network alterations and inflammatory processes, and exhibits marked mood sensitivity: emotional stress frequently exacerbates spasms, and imaging shows increased amygdala activation and altered connectivity with the motor cortex [14,15]. All HFS patients in our cohort were idiopathic, lacking identifiable peripheral lesions. The observation that BoNT-A improved mood in these cases strengthens the hypothesis that its affective effects are centrally mediated, likely through modulation of sensorimotor–limbic circuits. These findings argue against a purely peripheral explanation based on reduced nerve hyperactivity, motor symptom correction, or improvements in social and functional impairment. This aligns idiopathic HFS with dystonia in terms of potential central mechanisms contributing to motor hyperexcitability and emotional dysregulation, as suggested by neuroimaging evidence of structural and functional amygdala abnormalities in HFS patients [14].

### 3.3. Mechanistic Considerations: How BoNT-A Might Influence Mood

Although BoNT-A’s well-known mechanism involves cleavage of SNAP-25 at the presynaptic terminal, blocking acetylcholine release and inducing temporary chemodenervation of targeted muscles [35], a substantial body of evidence indicates that its action is not purely peripheral [36]. Because SNAP-25 is also a key component of synaptic vesicle fusion in central neurons, its cleavage (whether occurring through altered afferent input or potential retrograde transport) may modulate neurotransmitter release within corticostriatal and limbic circuits [37]. Such modulation provides a biologically plausible mechanism through which peripheral BoNT-A administration could exert central neuromodulatory effects [11], with downstream consequences relevant to mood regulation, as supported by recent clinical findings [38,39].

Altered sensory feedback following BoNT-A injections may recalibrate sensorimotor integration [12]. Dystonic patients exhibit excessive muscle spindle afferent activity and abnormal proprioceptive processing; by reducing aberrant muscle contraction, BoNT-A normalizes afferent signaling to the central nervous system [40,41]. This may, in turn, modulate the excitability of specific limbic–motor pathways, including projections between the basal ganglia, amygdala, anterior cingulate cortex, and supplementary motor area, which are known to integrate affective and motor processing, thereby indirectly improving emotional regulation.

From another perspective, retrograde transport of BoNT-A or its signaling fragments along axons has been demonstrated in animal models [11]. Although the extent of such transport in humans remains debated, studies have detected cleaved SNAP-25 in central neurons following peripheral administration, suggesting potential central synaptic modulation [42]. As reviewed by Luvisetto [12], accumulating preclinical and clinical evidence indicates that BoNT-A can reach central targets and modulate neuronal activity within the cortico-striatal and limbic circuits, supporting its broader neuromodulatory role beyond the neuromuscular junction.

Neuroimaging data from psychiatric studies and from aesthetic research involving glabellar BoNT-A injections in healthy individuals provide compelling evidence for central effects, demonstrating altered activity in limbic and prefrontal regions. Wollmer et al. [17] and Schulze et al. [43] showed that BoNT-A injections into the glabellar region reduce amygdala reactivity to negative stimuli and improve depressive symptoms in both clinically depressed and cosmetically treated individuals. Meta-analyses of randomized controlled trials confirm that these mood benefits exceed placebo response, implying a true neurobiological effect rather than a cosmetic or psychosocial artifact [44]. The “facial feedback” hypothesis offers one explanatory model by reducing proprioceptive input associated with negative facial expressions. BoNT-A may attenuate limbic activation patterns linked to sadness or anxiety [13,45]. Our findings extend these observations beyond the glabellar region. The consistent improvement in mood and anxiety among CD, BSP, and HFS patients (including those without any facial injections) suggests that BoNT-A’s affective benefits cannot be fully explained by facial feedback or appearance changes alone, but rather by modulatory effects on central affective networks through mechanisms that include altered afferent input, network-level plasticity, and possibly direct central action.

Beyond synaptic and network-level mechanisms, BoNT-A may also exert central effects through modulation of neuroinflammatory pathways. Preclinical studies demonstrate that BoNT-A reduces the release of pro-inflammatory mediators such as substance P, CGRP, and glutamate, attenuates microglial activation, and dampens peripheral and central sensitization [46]. These anti-inflammatory actions may contribute to improved emotional regulation, given the well-established link between neuroinflammation and mood disorders [47].

In addition, BoNT-A’s analgesic properties mediated through inhibition of nociceptive neurotransmitters, reduction in peripheral inflammation, and decreased central sensitization may further support mood improvement. Chronic pain is a major driver of anxiety and depressive symptoms in dystonia and hemifacial spasm; thus, BoNT-A-induced pain relief likely interacts synergistically with its neuromodulatory effects to enhance overall affective outcomes [46,48].

### 3.4. Integrative Model: Linking Diverse Pathways

Taken together, these findings suggest that BoNT-A exerts its non-motor effects through multiple, interacting pathways: by normalizing aberrant sensorimotor feedback [40], reducing excessive afferent drive to cortico-limbic networks, exerting possible central neuromodulatory actions via retrograde or trans-synaptic signaling [36], disrupting maladaptive emotion-motor coupling through reduced proprioceptive input from facial muscles [13,45], and finally by enhancing mood indirectly through psychosocial mechanisms such as improved self-image and relief from disabling motor symptoms [44,45]. This integrative framework explains why mood improvement was observed across conditions with distinct etiologies (dystonic, peripheral, or psychiatric) and why it was independent of BoNT-A dose or injection site in our study.

## 4. Limitations

Our study has several limitations-it includes modest sample size, small BSP subgroup, single-center design, reliance on self-report scales without formal psychiatric diagnostic interviews, heterogeneous injection protocols, and lack of objective neurophysiologic or neuroimaging correlates. Future research should build on these findings by including larger, carefully stratified patient groups and applying advanced neuroimaging methods. Such studies could help differentiate how much of BoNT-A’s mood benefit stems from its direct central effects on brain networks, changes in sensorimotor feedback or from the psychosocial relief that comes with improved motor control. Understanding these contributions will be essential to refining both neurological and psychiatric uses of BoNT-A.

## 5. Conclusions

Our findings indicate that BoNT-A’s mood-modulating effects extend beyond limbic (amygdala-based) pathways, likely engaging broader sensorimotor-limbic and cortico-striato-thalamo-cortical circuits. By bridging motor and emotional domains, BoNT-A offers a unique window into the shared neural substrates of movement and affect. Understanding BoNT-A’s central and network-level effects could reshape therapeutic strategies for both neurological and psychiatric disorders. Future studies combining longitudinal functional imaging, neurophysiological markers and detailed affective assessment could clarify whether specific neural circuits (e.g., amygdala-prefrontal or cerebellar-limbic pathways) mediate these benefits. Moreover, clarifying whether repeated BoNT-A treatments induce long-term plastic changes in emotional processing could have significant implications for personalized treatment of mood disorders.

## 6. Materials and Methods

Our cross-sectional single-center study gathered 30 participants with idiopathic CD, 22 with hemifacial spasm and 9 patients with blepharospasm from the Department of Neurology and Ophthalmology, University Hospital Osijek, Osijek, Croatia. The exclusion criteria were secondary cervical dystonias and recently diagnosed dementia, use of drugs that could affect cognitive functions or induce iatrogenic movement disorders, while for the HFS group, only idiopathic forms were included. Signed informed consent was obtained from all participants.

Baseline demographic characteristics of the study population are presented in Table 4. The three diagnostic groups (cervical dystonia, hemifacial spasm, and blepharospasm) did not differ significantly in age or gender distribution.

1. Clinical Assessments
  1.1. Motor Symptoms
    Modified Tsui Scale (TSUI) and Toronto Western Spasmodic Torticollis Rating Scale (TWSTRS) were used for cervical dystonia [49,50];
    Jankovic Rating Scale (JRS) was applied for blepharospasm and hemifacial spasm [51];
    TWSTRS questionnaire additionally assessed pain and disability in cervical dystonia patients
  1.2. Psychiatric and Cognitive Symptoms
    Beck Anxiety Inventory (BAI) and Beck Depression Inventory (BDI-II) were used across all groups [52,53];
    Mini-Mental State Examination (MMSE) confirmed absence of cognitive decline [54].
2. Study Design
  Motor, pain/disability, and psychiatric symptoms were assessed at two time points: before and three weeks after BoNT-A treatment. Subjects were enrolled only after the effect of prior therapy had subsided (≥3 months after last BoNT-A administration).
3. Botulinum Neurotoxin Administration
  BoNT-A administration scheme was individualized according to patient symptoms.
  Injection patterns followed established clinical guidelines for cervical dystonia, blepharospasm, and hemifacial spasm, with individualization based on symptom distribution and prior treatment response [55,56]. No additional manuals or stencils were used, as these protocols are standardized and routinely applied in clinical practice
  IncobotulinumtoxinA (INCO; Merz Pharma GmbH & Co. KGaA, Frankfurt/Main, Germany) was used. The toxin was reconstituted with sodium chloride 9 mg/mL (0.9%) solution (1 mL per 100 U vial or 0.5 mL per 50 U vial) using a 20–27 G short-bevel sterile needle. Each patient received 40–200 units of incoBoNT-A. Injections were predominantly performed by neurology specialists, with five blepharospasm cases treated by an ophthalmologist.

## Figures and Tables

**Table 1:** Comparison of observed scales’ values before and after botulinum toxin application.

	N	Before Application	After Application	*p*
BDI-II	61	5 (1.5–8)	3 (0–6)	<0.001 *
BAI	61	8 (3–15.5)	4 (1–11)	<0.001 *
TSUI total	30	7.5 (3.98)	5.17 (2.97)	0.001 ^#^
TWSTRS severity	30	13.9 (7.53)	8.47 (7.6)	0.006 ^#^
TWSTRS pain	30	7.49 (4.2)	4.57 (3.72)	0.001 ^#^
JRS	31	6 (6–8)	1 (0–2)	<0.001 *

BDI-II—Beck Depression Inventory-II; BAI—Beck Anxiety Inventory; TSUI—Modified Tsui Scale; TWSTRS—Toronto Western Spasmodic Torticollis Rating Scale. BDI-II and BAI scores are presented as median (interquartile range) due to non-normal distribution. TSUI and TWSTRS scores are presented as mean ± standard deviation, reflecting normal distribution. Statistical comparisons were performed using the Wilcoxon Signed Ranks Test * and # Paired-Samples T Test.

**Table 2:** Correlation between botulinum toxin dosage and mood changes after treatment.

	Condition	*N*	Before Application	After Application	*p*
BDI-II	Cervical dystonia	30	5 (1.75–8.5)	3 (0–6)	0.12 *
	Hemifacial spasm	22	3 (1–7.5)	2 (0–6)	
	Blepharospasm	9	7 (3–9)	3 (05–6.5)	
BAI	Cervical dystonia	30	8 (4.75–22)	4.5 (1.75–16.25)	0.18 *
	Hemifacial spasm	22	5.5 (1.75–12.75)	4 (0–8)	
	Blepharospasm	9	9 (6–14.5)	3 (1–8)	

* *N* = number of patients per group; *p* = *p*-value of the statistical test applied, Beck Anxiety Inventory (BAI), Beck Depression Inventory (BDI-II). Values are presented as median (interquartile range) before and three weeks after BoNT-A treatment.

**Table 3:** Differences in changes in BDI-II and BAI scores after botulinum toxin application in regard to glabellar application.

	Application	N	Before Application	After Application	*p*
BDI-II	Glabellar region	23	4 (1–7)	2 (0–6)	0.92
	Without glabellar region	38	5 (2–9.25)	3 (0–6)	
BAI	Glabellar region	23	8 (3–11)	3 (0–7)	0.40
	Without glabellar region	38	8 (3.75–20.25)	4.5 (1.75–14.25)	

Values are presented as median (interquartile range) before and three weeks after BoNT-A treatment. BDI-II—Beck Depression Inventory-II; BAI—Beck Anxiety Inventory; N—number of patients per group. *p*—*p*-value of Student’s T-test. “Glabellar region” refers to patients who received BoNT-A injections in the glabellar area. “Without glabellar region” refers to patients treated in other clinically relevant regions (e.g., periorbicular, perioral, cheek, cervical, or cranial muscles, depending on diagnosis).

**Table 4:** Baseline demographic characteristics of study participants.

Diagnostic Group	N	Age (Mean ± SD)	Gender (M/F)
Cervical dystonia (CD)	30	59.2 ± 12.2	8/22
Hemifacial spasm (HFS)	22	63.6 ± 13.7	4/18
Blepharospasm (BSP)	9	62.0 ± 10.4	2/7

Age is presented as mean ± standard deviation. Gender distribution is shown as male/female.

## Data Availability

The original contributions presented in this study are included in the article. Further inquiries can be directed to the corresponding author.

## Abbreviations

The following abbreviations are used in this manuscript:

BoNT-A	Botulinum toxin type A
CD	Cervical dystonia
HFS	Hemifacial spasm
BDI-II	Beck Depression Inventory-II
BAI	Beck Anxiety Inventory
TSUI	Modified Tsui Scale
TWSTRS	Toronto Western Spasmodic Torticollis Rating Scale
JRS	Jankovic Rating Scale
MMSE	Mini–Mental State Examination

## References

[1] Albanese A., Bhatia K., Bressman S.B., Delong M.R., Fahn S., Fung V.S., Hallett M., Jankovic J., Jinnah H.A., Klein C. (2013). Phenomenology and classification of dystonia: A consensus update. Mov. Disord..

[2] Sugar D., Pirio Richardson S. (2025). Treating non-motor symptoms in dystonia: A systematic review. Dystonia.

[3] Bailey G.A., Martin E., Peall K.J. (2022). Cognitive and Neuropsychiatric Impairment in Dystonia. Curr. Neurol. Neurosci. Rep..

[4] Novaretti N., Cunha A.L.N., Bezerra T.C., Pena Pereira M.A., de Oliveira D.S., Macruz Brito M.M.C., Pimentel A.V., Brozinga T.R., Foss M.P., Tumas V. (2019). The Prevalence and Correlation of Non-motor Symptoms in Adult Patients with Idiopathic Focal or Segmental Dystonia. Tremor Other Hyperkinetic Mov..

[5] Kuric T., Popović Z., Matosa S., Sadikov A., Groznik V., Georgiev D., Gerbasi A., Kragujevic J., Zubonja T.M., Dupan Z.K. (2024). Memory-Guided Saccades and Non-Motor Symptoms Improve after Botulinum Toxin Therapy in Cervical Dystonia. J. Clin. Med..

[6] Guadagni V., Burles F., Callahan B.L., Iaria G., Martino D. (2025). Functional connectivity of brain areas related to social cognition and anxiety in cervical dystonia. Dystonia.

[7] Wang X., Hu W., Wang H., Gao D., Liu Y., Zhang X., Jiang Y., Mo J., Meng F., Zhang K. (2022). Altered Structural Brain Network Topology in Patients with Primary Craniocervical Dystonia. Front. Neurol..

[8] Zoons E., Dijkgraaf M.G., Dijk J.M., van Schaik I.N., Tijssen M.A. (2012). Botulinum toxin as treatment for focal dystonia: A systematic review of the pharmaco-therapeutic and pharmaco-economic value. J. Neurol..

[9] Weise D., Weise C.M., Naumann M. (2019). Central Effects of Botulinum Neurotoxin—Evidence from Human Studies. Toxins.

[10] Gilman Kuric T., Popović Z., Tomić S., Kragujević J., Mirošević Zubonja T., Pučić T., Petek Erić A., Kuric I. (2023). Psychiatric symptoms in women with focal cervical dystonia—Exploring new pathophysiological pathways. Arch. Psychiatry Res. Int. J. Psychiatry Relat. Sci..

[11] Antonucci F., Rossi C., Gianfranceschi L., Rossetto O., Caleo M. (2008). Long-distance retrograde effects of botulinum neurotoxin A. J. Neurosci..

[12] Luvisetto S. (2021). Botulinum Neurotoxins in Central Nervous System: An Overview from Animal Models to Human Therapy. Toxins.

[13] Hanayama H., Tada J., Terashi H. (2024). Botulinum Toxin A Therapy for Glabellar Lines Improves Emotional States: Evaluation of 47 Cases without Mental Disorders. J. Plast. Reconstr. Surg..

[14] Brodoehl S., Wagner F., Prell T., Klingner C., Witte O.W., Günther A. (2019). Cause or effect: Altered brain and network activity in cervical dystonia is partially normalized by botulinum toxin treatment. Neuroimage Clin..

[15] Stark S., Stark C., Wong B., Brin M.F. (2023). Modulation of amygdala activity for emotional faces due to botulinum toxin type A injections that prevent frowning. Sci. Rep..

[16] Bress K.S., Cascio C.J. (2024). Sensorimotor regulation of facial expression—An untouched frontier. Neurosci. Biobehav. Rev..

[17] Wollmer M.A., Magid M., Krüger T.H.C., Finzi E. (2022). Treatment of Depression with Botulinum Toxin. Toxins.

[18] Finzi E., Rosenthal N.E. (2014). Treatment of depression with onabotulinumtoxinA: A randomized, double-blind, placebo-controlled trial. J. Psychiatr. Res..

[19] Xu H., Guo C., Li H., Gao L., Zhang M., Wang Y. (2019). Structural and Functional Amygdala Abnormalities in Hemifacial Spasm. Front. Neurol..

[20] Gao W., Yang D., Zhang Z., Du L., Liu B., Liu J., Chen Y., Wang Y., Liu X., Yang A. (2021). Altered cortical–striatal network in patients with hemifacial spasm. Front. Hum. Neurosci..

[21] Chen M., Yang M., Zhou W.P., Li S.T. (2019). Preliminary Study on the Relationship Between Inflammation and Hemifacial Spasm. World Neurosurg..

[22] Nevrlý M., Hluštík P., Hok P., Otruba P., Tüdös Z., Kaňovský P. (2018). Changes in sensorimotor network activation after botulinum toxin type A injections in patients with cervical dystonia: A functional MRI study. Exp. Brain Res..

[23] Hennenlotter A., Dresel C., Castrop F., Ceballos-Baumann A.O., Wohlschläger A.M., Haslinger B. (2009). The link between facial feedback and neural activity within central circuitries of emotion—New insights from botulinum toxin-induced denervation of frown muscles. Cereb Cortex.

[24] Magid M., Reichenberg J.S., Poth P.E., Robertson H.T., LaViolette A.K., Kruger T.H., Wollmer M.A. (2014). Treatment of major depres-sive disorder using botulinum toxin A: A 24-week randomized, double-blind, placebo-controlled study. J. Clin. Psychiatry.

[25] Brüggemann N. (2021). Contemporary functional neuroanatomy and pathophysiology of dystonia. J. Neural Transm..

[26] Jinnah H.A., Neychev V., Hess E.J. (2017). The Anatomical Basis for Dystonia: The Motor Network Model. Tremor Other Hyperkinetic Mov..

[27] Giannì C., Pasqua G., Ferrazzano G., Tommasin S., De Bartolo M.I., Petsas N., Belvisi D., Conte A., Berardelli A., Pantano P. (2022). Focal Dystonia: Functional Connectivity Changes in Cerebellar-Basal Ganglia-Cortical Circuit and Preserved Global Functional Architecture. Neurology.

[28] Filip P., Gallea C., Lehéricy S., Bertasi E., Popa T., Mareček R., Lungu O.V., Kašpárek T., Vaníček J., Bareš M. (2017). Disruption in cerebellar and basal ganglia networks during a visuospatial task in cervical dystonia. Mov. Disord..

[29] Stamelou M., Edwards M.J., Hallett M., Bhatia K.P. (2012). The non-motor syndrome of primary dystonia: Clinical and pathophysiological implications. Brain.

[30] Yadav R., Ray S., Pal P. (2020). Non-Motor Symptoms in Cervical Dystonia: A Review. Ann. Indian Acad. Neurol..

[31] Kuyper D.J., Parra V., Aerts S., Okun M.S., Kluger B.M. (2011). Nonmotor manifestations of dystonia: A systematic review. Mov. Disord..

[32] Sasikumar S., Strafella A.P. (2021). The neuroimaging evidence of brain abnormalities in functional movement disorders. Brain.

[33] Argyelan M., Carbon M., Niethammer M., Ulug A.M., Voss H.U., Bressman S.B., Dhawan V., Eidelberg D. (2009). Cerebellothalamocortical connectivity regulates penetrance in dystonia. J. Neurosci..

[34] Jesuthasan A., Natalwala A., Davagnanam I., Saifee T., Zrinzo L. (2025). Hemifacial spasm: An update on pathophysiology, investigations and management. J. Neurol..

[35] Blasi J., Chapman E.R., Link E., Binz T., Yamasaki S., De Camilli P., Südhof T.C., Niemann H., Jahn R. (1993). Botulinum neurotoxin A selectively cleaves the synaptic protein SNAP-25. Nature.

[36] Hok P., Veverka T., Hluštík P., Nevrlý M., Kaňovský P. (2021). The Central Effects of Botulinum Toxin in Dystonia and Spasticity. Toxins.

[37] Antonucci F., Corradini I., Fossati G., Tomasoni R., Menna E., Matteoli M. (2016). SNAP-25, a Known Presynaptic Protein with Emerging Postsynaptic Functions. Front. Synaptic. Neurosci..

[38] De la Torre Canales G., Poluha R.L., Bonjardim L.R., Ernberg M., Conti P.C.R. (2024). Botulinum toxin-A effects on pain, somatosensory and psychosocial features of patients with refractory masticatory myofascial pain: A randomized double-blind clinical trial. Sci. Rep..

[39] Marfoli A., Mameli F., Aiello E.N., Ruggiero F., Sandi A., Mellace D., Curti B., Vimercati R., Poletti B., Ticozzi N. (2024). Does botulinum toxin affect psycho-social aspects in dystonia?. J. Neural Transm..

[40] Avanzino L., Fiorio M. (2014). Proprioceptive dysfunction in focal dystonia: From experimental evidence to rehabilitation strategies. Front. Hum. Neurosci..

[41] Quartarone A., Hallett M. (2013). Emerging concepts in the physiological basis of dystonia. Mov. Disord..

[42] Restani L., Giribaldi F., Manich M., Bercsenyi K., Menendez G., Rossetto O., Caleo M., Schiavo G. (2012). Botulinum neurotoxins A and E undergo retrograde axonal transport in primary motor neurons. PLoS Pathog..

[43] Schulze J., Neumann I., Magid M., Finzi E., Sinke C., Wollmer M.A., Krüger T.H.C. (2021). Botulinum toxin for the management of depression: An updated review of the evidence and meta-analysis. J. Psychiatr. Res..

[44] Qian H., Shao F., Lenahan C., Shao A., Li Y. (2020). Efficacy and Safety of Botulinum Toxin vs. Placebo in Depression: A Systematic Review and Meta-Analysis of Randomized Controlled Trials. Front. Psychiatry.

[45] Kim M.J., Neta M., Davis F.C., Ruberry E.J., Dinescu D., Heatherton T.F., Stotland M.A., Whalen P.J. (2014). Botulinum toxin-induced facial muscle paralysis affects amygdala responses to the perception of emotional expressions: Preliminary findings from an A-B-A design. Biol. Mood Anxiety Disord.

[46] Park J., Park H.J. (2017). Botulinum Toxin for the Treatment of Neuropathic Pain. Toxins.

[47] Miller A.H., Raison C.L. (2016). The role of inflammation in depression: From evolutionary imperative to modern treatment target. Nat. Rev. Immunol..

[48] Gureje O., Von Korff M., Simon G.E., Gater R. (1998). Persistent pain and well-being: A World Health Organization study in primary care. JAMA.

[49] Consky E. (1990). The Toronto Western Spasmodic Torticollis Rating Scale (TWSTRS): Assessment of validity and inter-rater reliability. Neurology.

[50] Tsui J.K., Eisen A., Stoessl A.J., Calne S., Calne D.B. (1986). Double-blind study of botulinum toxin in spasmodic torticollis. Lancet.

[51] Titi-Lartey O.A., Patel B.C. (2025). Benign Essential Blepharospasm. StatPearls.

[52] Beck A.T., Ward C.H., Mendelson M., Mock J., Erbaugh J. (1961). An inventory for measuring depression. Arch. Gen. Psychiatry.

[53] Beck A.T., Epstein N., Brown G., Steer R.A. (1988). An inventory for measuring clinical anxiety: Psychometric properties. J. Consult. Clin. Psychol..

[54] Folstein M.F., Folstein S.E., McHugh P.R. (1975). “Mini-mental state”: A practical method for grading the cognitive state of patients for the clinician. J. Psychiatr. Res..

[55] Albanese A., Asmus F., Bhatia K.P., Elia A.E., Elibol B., Filippini G., Gasser T., Krauss J.K., Nardocci N., Newton A. (2011). EFNS guidelines on diagnosis and treatment of primary dystonias. Eur. J. Neurol..

[56] Kenney C., Jankovic J. (2008). Botulinum toxin in the treatment of blepharospasm and hemifacial spasm. J. Neural Transm..

